# Maintaining Low BCR-ABL Signaling Output to Restrict CML Progression and Enable Persistence

**DOI:** 10.1007/s11899-013-0196-8

**Published:** 2014-02-06

**Authors:** Andreas Burchert

**Affiliations:** Hematology, Oncology and Immunology, Philipps University Marburg, University Hospital Gießen and Marburg (UKGM), Campus Marburg, 35043 Marburg, Germany

**Keywords:** CML, Imatinib, BCR-ABL, Persistence, Kinase inhibitors

## Abstract

Deregulated BCR-ABL oncogenic activity leads to transformation, oncogene addiction and drives disease progression in chronic myeloid leukemia (CML). Inhibition of BCR-ABL using Abl-specific kinase inhibitors (TKI) such as imatinib induces remarkable clinical responses. However, approximately only less than 15 % of all chronic-phase CML patients will remain relapse-free after discontinuation of imatinib in deep molecular remission. It is not well understood why persisting CML cells survive under TKI therapy without developing clonal evolution and frank TKI resistance. BCR-ABL expression level may be critically involved. Whereas higher BCR-ABL expression has been described as a pre-requisite for malignant CML stem cell transformation and CML progression to blast crisis, recent evidence suggests that during persistence TKI select for CML precursors with low BCR-ABL expression. Genetic, translational and clinical evidence is discussed to suggest that TKI-induced maintenance of low BCR-ABL signaling output may be potently tumor suppressive, because it abrogates oncogenic addiction.

## Introduction

Neoplastic transformation is considered to involve a sequence of independent mutations, which activate oncogenes or inactivate tumor suppressor genes [1, 2]. In contrast, chronic myeloid leukemia (CML) transformation is commonly viewed to originate from a single, causative genetic mutation, *Bcr-Abl*, which emerges at the level of a hematopoietic stem cell [3–6]. There is evidence that increased BCR-ABL expression contributes to progression from chronic to accelerated phase and blast crisis [7] (reviewed in [8]). Supposedly, also the conversion of BCR-ABL-positive, pre-malignant stem cells into malignant CML stem cells (reviewed in [2]) may involve rising BCR-ABL levels. BCR-ABL tyrosine kinase inhibitors (TKI) induce durable molecular remissions and potently protect from progression into accelerated phase and blast crisis [9–12]. This provides indirect evidence that TKI treatment prevents the evolution of BCR-ABL overexpressing clones. In other words, TKI could be tumor suppressive by preventing spontaneous emergence of clones with high BCR-ABL signaling output.

On the other hand, TKI therapy rarely leads to CML stem cell eradication (reviewed in [13]). BCR-ABL positive progenies and stem cells persist [14–16•] despite the fact that their BCR-ABL kinase activity is potently inhibited [17–19]. Since recurrences after TKI cessation are always ABL-TKI sensitive [20–22], the underlying nature of CML stem cell persistence is evidently BCR-ABL-independent. Selection of persisting clones with low BCR-ABL signaling output has been suggested as an underlying mechanism of CML persistence by preventing BCR-ABL addiction and thus TKI sensitivity [16•, 23].

Together, several lines of evidence suggest that whereas increased BCR-ABL dosage controls CML transformation and progression [7, 8], TKI treatment reverts this by suppressing survival of cells with high BCR-ABL signaling output [16•, 24, 25]. This effect of TKI could be an important prerequisite for long-term disease control in chronic phase.

## BCR-ABL-Mediated Stem Cell Transformation: Dose Really Matters

In vitro replating results and in vivo transplantation experiments support the notion that BCR-ABL is a comparably weakly transforming oncogene. Unlike, for example, AML oncogenes such as MOZ-TIF2 or MLL-ENL, BCR-ABL is incapable of conferring self-renewal to non-stem cells [26] unless additional mutations are also present [27–32]. Translational evidence shows that BCR-ABL mRNA can frequently be detected also in healthy individuals who will never develop CML [33, 34], suggesting that BCR-ABL alone may not suffice to transform directly. On the other hand, BCR-ABL transduction-transplantation models [3, 6, 35, 36] and transgenic CML models [37–39], including conditional transgenic CML mice models, in which BCR-ABL expression was targeted to stem cells [40, 41], suggest that BCR-ABL is sufficient to initiate a rapidly fatal CML-like myeloproliferation. However, unlike in the chronic phase of the human disease, BCR-ABL expression in transgenic CML is artificial: BCR-ABL is overexpressed—often from multiple BCR-ABL copies and active promoters located outside of the endogenous *Bcr* locus. In contrast, when only one or two copies of BCR-ABL^p210^ are expressed from the endogenous promoter in the *Bcr* locus, transgenic BCR-ABL animals do not develop CML during their entire lifetime [42•]. In fact, BCR-ABL-positive hematopoiesis in these mice behaved completely normal with the exception of a slightly better engraftment potential. This was due to faster proliferation, not increased stem cell self-renewal. BCR-ABL-positive hematopoiesis was also not BCR-ABL-addicted and consequently not TKI sensitive [42•]. Authors concluded that BCR-ABL on its does not transform, but requires cooperating mutations. However, this conclusion still remains to be proven. Alternatively, the time needed to select for high BCR-ABL levels could be beyond the lifespan of a mouse. Moreover, additional mutations—instead of being directly cooperative with BCR-ABL in transformation as suggested—might be required to enable tolerance against high BCR-ABL expression levels (see section below: barriers against transformation). There is precedence for this genetic concept from Myc-dependent tumor models. Whereas induction of *Myc* causes tumorigenesis, subsequent Myc repression rarely leads to a complete elimination of the tumor. Tumors eventually become Myc-independent [43–45]. This means, that although an oncogene such as Myc (or BCR-ABL) can be instrumental for the initiation of tumorigenesis, secondary genetic or epigenetic changes may be required to tolerate elevated oncogenic stress and subsequently also allow independence from the causative oncogene [46]. This has been demonstrated for the emergence of *Kras2* mutations in Myc-dependent mouse mammary tumors [44].

However, before oncogenic signaling stress causes transformation, it usually engages tumor suppressive barriers. It is important to discuss, therefore, barriers against transformation in hematopoietic stem cells, when they are activated and how they fail.

## Tumor Suppressive Mechanisms in CML

### General Barriers Against Transformation

Two major tumorigenesis barriers exist. Oncogene-induced DNA damage response (DDR) [47–50] (reviewed in [51]) is characterized by expression of oncogene-induced DNA damage checkpoints such as ATM, ATR, γH2AX and chk2 [47, 52]. Increased expression of the tumor suppressors p16^INK4A^ and p19^Arf^ has been shown to act as an alternative tumor suppressive barrier governed by oncogenic signal flux [53–56]. Both barriers, DDR and induction of p16^INK4A^ and p19^Arf,^ converge at the level of p53 and stabilize its expression to restrain transformation by elicitation of apoptosis, senescence or differentiation (for review: [51, 54, 57]). Mutations in both pathways breach off these barriers, rescue oncogene-induced proliferation and allow malignancy to develop.

### Engaging Arf-p53 by BCR-ABL in Stem Cells

It is remarkable that p53-inactivating mutations—one of the most common mutations in tumors – are absent in chronic phase of CML. Even CML blast crisis patients relatively seldom acquire p53 mutations (20–25 %) [58]. Indeed, p53 remains functional upon appropriate challenge in most patients in chronic and progressed phases of CML [59, 60, 61]. This suggests a lack of genetic pressure to mutate the p53 checkpoint during BCR-ABL-induced stem cell transformation. What are possible reasons for this?

**Fig. 1:**
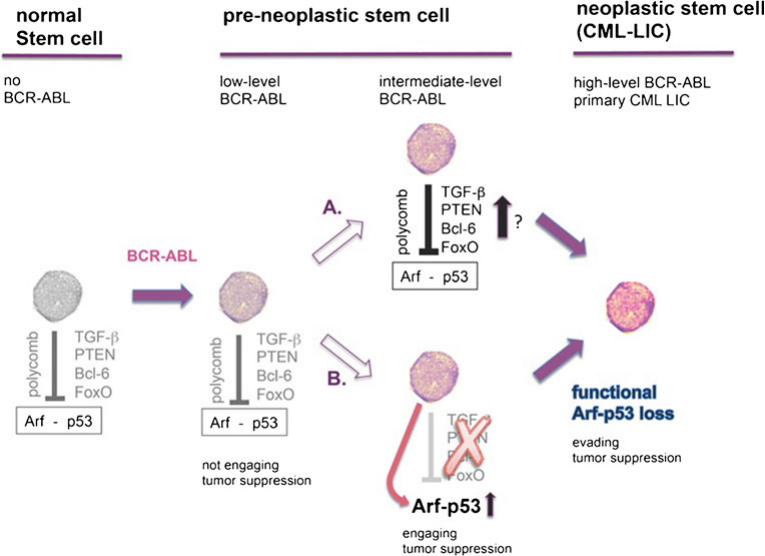
BCR-ABL levels govern engagement and evasion of tumor suppression in hematopoietic precursors, thereby controlling CML transformation. In normal hematopoietic stem cells, p53 negatively regulates self-renewal and p53 pathway activation is suppressed (left). During CML evolution, increasing BCR-ABL expression level must be tolerated (middle) by adjustment of pathways that suppress Arf-p53 activation (arm A), or by alternatively mutating the Arf-p53 checkpoint to resolve a pressure on this pathway (arm B). The consequence of both scenarios (arm A or B) would be tolerance of high-level BCR-ABL, which results in BCR-ABL overexpression induced transformation and CML progression

First, CML arises from a normal pluripotent stem cell, which lacks expression of relevant functional levels of p53, because in stem cells, p53 negatively regulates self-renewability, quiescence [62–65] and pluripotency by reprogramming [66]. Secondly, polycomb repressor complexes epigenetically silence the Cdkn2a/b gene cluster (encoding INK-4A/ARF) in hematopoietic stem cells. This ameliorates the Arf-HDM2-p53 pathway and explains the failure to select for CDKN2A deletion in the presence of BCR-ABL [67–69] (Fig. 1). Third, BCR-ABL signaling has different consequences in stem versus progenitor cells. For example, BCR-ABL activates PI3K-Akt signaling and thus inactivates FoxO transcription factors in CML progenitors. This results in apoptosis inhibition and proliferation [70–73•]. In contrast, in stem cells, BCR-ABL-dependent Akt pathway activation is repressed by TGF-beta signaling, which limits oncogenic stress [72]. Bcl-6 – as a downstream target of FoxO3 has also been demonstrated to bind to and repress Arf and p53 promoters in BCR-ABL-positive ALL [74] and in CML [73•], which also compromises the p53 checkpoint (Fig. 1). Finally, reduced p53 function was shown to result from BCR-ABL-induced overexpression of the deacetylase SIRT1, which selectively increases survival of CML stem cells [59, 75]. Together, several factors contribute to BCR-ABL stress tolerance in hematopoietic stem cells by inhibition of an Arf-p53 response.

BCR-ABL is an oncogenic driver mutation [1]. Driver mutations are not directly transforming, but initially induce only mild increases in cell proliferation, e.g., on the order of 0.4 % growth difference between cell birth and cell death. However, it has been estimated that this mild proliferative advantage over normal cells leads within years to massive expansion of driver mutation-positive cells, which are amenable to acquire secondary hits [76] and tolerate higher oncogene levels (Fig. 1). Indeed, in the mouse model by Foley et al., BCR-ABL did not transform into CML, but conferred a growth advantage of BCR-ABL-positive over normal hematopoiesis [42•].

### BCR-ABL-Triggered Progression in Non-Stem Cells

Mechanisms of CML progression in progenitors have been well established [8, 77] and are not in the focus of this review. A myriad of secondary, cooperating genetic mutations [8, 77] and gene expression changes [78] have been shown to be associated with the reprogramming of progenitors into secondary leukemia initiating cells. Some of these mutations obviously directly or indirectly disrupt the Arf-p53 tumor surveillance pathway highlighting its importance during progression. Prominent examples would be the deletion of the CDKN2A locus (deleting INK4A and ARF) [30, 69, 79], p53 mutations [58], β-Catenin overexpression [28], by conferring self-renewal and helping to maintain polycomb-mediated silencing of CDKN2A/B, as well as other mutations.

## CML Transformation: Escaping Tumor Suppressive Barriers

Compelling evidence suggests that distinct thresholds of oncogenic Ras- or Myc-signaling decisively control biologic output during tumorigenesis. Whereas low levels of oncogenic Ras and Myc suffice to drive proliferation, they are insufficient to engage tumor suppression via activating p53-INK4A-Arf-dependent tumor suppressive barriers [80, 81••, 82].

According to this concept and the data that have been discussed in the previous two sections, pre-malignant BCR-ABL-positive stem cells may initially tolerate BCR-ABL without engaging endogenous tumor suppression pathways (Fig. 1). However, the requirement to sustain such low BCR-ABL expression levels to prevent transformation will establish an intrinsic selection advantage favoring, exactly, BCR-ABL overexpression. BCR-ABL overexpression is subject to positive selection, because it confers increased aggressiveness to the evolving leukemia. There is also translational evidence for this in vivo: clonal heterogeneity of lower BCR-ABL expression level at diagnosis [16•] is followed by BCR-ABL overexpression as an undisputed hallmark of CML progression [8]. However, tolerance to raising BCR-ABL activity necessitates further erosion of the p53 tumor suppressor pathway (Fig. 1, arm A), or spontaneous mutagenesis inactivates the engaged p53 checkpoint (Fig. 1, arm B). Either way, the result would be tolerance of high-level of BCR-ABL and thus progression.

## CML Persistence: Limiting BCR-ABL Signaling Strength

The basis for clinical ABL kinase inhibitor responsiveness in CML is a strict survival dependence on the BCR-ABL kinase activity, so-called oncogene addiction [83]. The emergence of BCR-ABL kinase mutations enables survival in the presence of TKI and thus unequivocally proves addiction of the resistant clone to BCR-ABL. Notably, CML patients in stable molecular remission (MMR, MR^4^ or better) harbor BCR-ABL-positive residual disease [14–16•, 24, 25], but have a neglectable chance to develop TKI resistance or BCR-ABL kinase mutations. This excludes BCR-ABL addiction of persisting CML. There are a plethora of proposed mechanisms to explain persistence [13, 84]. However, due to a notorious difficulty to investigate rare BCR-ABL-positive residual clones, most of the suggested imatinib and TKI persistence mechanisms have been derived using cell lines or pre-therapeutic CD34^+^ CML samples, which may not exactly reflect in vivo regulations during long term persistence [8, 85–90].

**Fig. 2:**
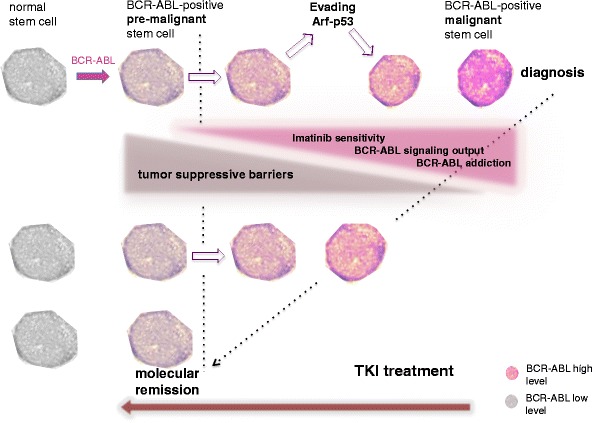
Suppression of high-level deregulated BCR-ABL-signaling intensity as a putative tumor suppressive mechanism of TKI. CML evolution is characterized by a continuous selection of high-level BCR-ABL-expressing clones, because high-level oncogene activity provides an increased aggressiveness and thus a growth advantage. BCR-ABL will engage intrinsic tumor suppressive pathways (such as Arf -p53) and cause a selective pressure against these pathways. Resolving this pressure by pathway mutagenesis will lead to CML progression. However, increasing BCR-ABL levels are also associated with strong BCR-ABL addiction and thus sensitivity to BCR-ABL kinase inhibitors such as imatinib. By depleting BCR-ABL-overexpressing clones and selecting for low-level BCR-ABL signaling output (indicated by decreasing red-staining intensity of nuclei), imatinib would counteract BCR-ABL addiction, and become tumor suppressive. It could be proposed that only TKI therapy-mediated suppression of BCR-ABL expression below a certain threshold (indicated as dotted line) may enable long-term persistence of residual CML clones

We have recently found that imatinib treatment shapes the BCR-ABL expression repertoire in patients from “high” in pre-therapeutic CD34^+^ CML clones to “low” during persistence [16•]. In the context of the presumed fundamental role that increasing BCR-ABL levels play to ultimately establish malignant CML stem cells, this finding suggests that imatinib treatment reverses CML leukemogenesis by leaving behind low level BCR-ABL expressing CML stem cells (Figs. 1 and 2). Studies on persisting BCR-ABL-positive long-term culture initiating cells (enriching for stem cells) supported our findings in a different patient population. It was demonstrated that low-level BCR-ABL expressing stem cells persist long-term under imatinib [24]. Modeling high versus low BCR-ABL expression in primary human CD34^+^ precursors and murine progenitors suggested that high-level BCR-ABL expression sensitizes to imatinib by induction of oncogene addiction [16•, 23] (Fig. 2).

In the context of the finding that imatinib selects for survival of clones with low-level deregulated BCR-ABL [16•] (Fig. 2), we suggested that potent and durable suppression of deregulated BCR-ABL activity is an important mechanism of tumor suppression by inhibiting oncogenic addiction. The recently published Australian TWISTER study, which shows that essentially all patients, who successfully discontinue imatinib will remain BCR-ABL DNA positive, may add another important aspect to this model [22]. Low-level BCR-ABL DNA detection could be also compatible with the presence not only of low BCR-ABL expressing malignant clones, but as well represent pre-malignant BCR-ABL positive populations.

## Potential Clinical Implications

1. Imatinib’s tumor suppressive role during persistence is mediated, at least in part, by its ability to induce potently and durably and maintain a low level of BCR-ABL oncogenic output. Patients failing to rapidly eradicate high-level BCR-ABL expressing clones (e.g., presumably mirrored by slow decline of BCR-ABL ratio), therefore, have a greater chance of developing resistance.
2. Once stable persistence of low-level BCR-ABL expression has been achieved (low BCR-ABL ratio), evolution of resistance is unlikely, because persisting disease is characterized by low oncogenic signaling output. If necessary due to side effects, less intensive ABL-TKI therapy may then control residual disease without risk of progression. The clinical implication would be that TKI therapy could be de-escalated once optimal molecular response has been documented.
3. An inclusion criterion for imatinib discontinuation trials was presence of a stable MR^4.5^. This is achieved by approximately 34 % of all CML-CP patients after 8 years [22]. Since residual disease is BCR-ABL-independent, it is not clear whether more potent Abl kinase inhibition will indeed increase the absolute number of patients that remain relapse-free after TKI discontinuation in stable MR^4.5^.
4. If confirmed in other oncogene-addicted cancers [91] and leukemias such as Flt3-ITD-dependent acute myeloid leukemia [92], suppression of high oncogene levels may be established as a general mechanism to predict effectiveness of kinase inhibitor therapy.

## References

[1] Vogelstein B, Papadopoulos N, Velculescu VE, Zhou S, Diaz LA, Kinzler KW (2013). Cancer genome landscapes. Science..

[2] Valent P, Bonnet D, De Maria R, Lapidot T, Copland M, Melo JV (2012). Cancer stem cell definitions and terminology: the devil is in the details. Nat. Rev. Cancer..

[3] Ben-Neriah Y, Daley GQ, Mes-Masson AM, Witte ON, Baltimore D (1986). The chronic myelogenous leukemia-specific P210 protein is the product of the bcr/abl hybrid gene. Science..

[4] Nowell PC, Hungerford DA (1961). Chromosome studies in human leukemia. II. Chronic granulocytic leukemia. J. Natl. Cancer Inst..

[5] Rowley JD (1973). Letter: a new consistent chromosomal abnormality in chronic myelogenous leukaemia identified by quinacrine fluorescence and Giemsa staining. Nature..

[6] Daley GQ, Van Etten RA, Baltimore D (1990). Induction of chronic myelogenous leukemia in mice by the P210bcr/abl gene of the Philadelphia chromosome. Science..

[7] Gaiger A, Henn T, Hörth E, Geissler K, Mitterbauer G, Maier-Dobersberger T (1995). Increase of bcr-abl chimeric mRNA expression in tumor cells of patients with chronic myeloid leukemia precedes disease progression. Blood..

[8] Perrotti D, Jamieson C, Goldman J, Skorski T (2010). Chronic myeloid leukemia: mechanisms of blastic transformation. J. Clin. Invest..

[9] Hochhaus A, O’Brien SG, Guilhot F, Druker BJ, Branford S, Foroni L (2009). Six-year follow-up of patients receiving imatinib for the first-line treatment of chronic myeloid leukemia. Leukemia..

[10] Hehlmann R, Lauseker M, Jung-Munkwitz S, Leitner A, Müller MC, Pletsch N (2011). Tolerability-adapted imatinib 800 mg/d versus 400 mg/d versus 400 mg/d plus interferon-α in newly diagnosed chronic myeloid leukemia. J. Clin. Oncol..

[11] Larson RA, Hochhaus A, Hughes TP, Clark RE, Etienne G, Kim DW (2012). Nilotinib vs imatinib in patients with newly diagnosed Philadelphia chromosome-positive chronic myeloid leukemia in chronic phase: ENESTnd 3-year follow-up. Leukemia..

[12] Kantarjian H, Shah NP, Hochhaus A, Cortes J, Shah S, Ayala M (2010). Dasatinib versus imatinib in newly diagnosed chronic-phase chronic myeloid leukemia. N. Engl. J. Med..

[13] O’Hare T, Zabriskie MS, Eiring AM, Deininger MW (2012). Pushing the limits of targeted therapy in chronic myeloid leukaemia. Nat. Rev. Cancer..

[14] Chu S, McDonald T, Lin A, Chakraborty S, Huang Q, Snyder DS (2011). Persistence of leukemia stem cells in chronic myelogenous leukemia patients in prolonged remission with imatinib treatment. Blood..

[15] Chomel J-C, Bonnet M-L, Sorel N, Bertrand A, Meunier M-C, Fichelson S (2011). Leukemic stem cell persistence in chronic myeloid leukemia patients with sustained undetectable molecular residual disease. Blood..

[CR16] Kumari A, Brendel C, Hochhaus A, Neubauer A, Burchert A (2012). Low BCR-ABL expression levels in hematopoietic precursor cells enable persistence of chronic myeloid leukemia under imatinib. Blood..

[17] Corbin AS, Agarwal A, Loriaux M, Cortes J, Deininger MW, Druker BJ (2011). Human chronic myeloid leukemia stem cells are insensitive to imatinib despite inhibition of BCR-ABL activity. J. Clin. Invest..

[18] Hamilton A, Helgason GV, Schemionek M, Zhang B, Myssina S, Allan EK (2012). Chronic myeloid leukemia stem cells are not dependent on Bcr-Abl kinase activity for their survival. Blood..

[19] Neviani P, Harb JG, Oaks JJ, Santhanam R, Walker CJ, Ellis JJ, et al. PP2A-activating drugs selectively eradicate TKI-resistant chronic myeloid leukemic stem cells. J. Clin. Invest. 2013. 10.1172/JCI68951 PMC3784537 23999433

[20] Cortes J, O’Brien S, Kantarjian H (2004). Discontinuation of imatinib therapy after achieving a molecular response. Blood..

[21] Mahon F-X, Réa D, Guilhot J, Guilhot F, Huguet F, Nicolini F (2010). Discontinuation of imatinib in patients with chronic myeloid leukaemia who have maintained complete molecular remission for at least 2 years: the prospective, multicentre Stop Imatinib (STIM) trial. Lancet Oncol..

[22] Ross DM, Branford S, Seymour JF, Schwarer AP, Arthur C, Yeung DT (2013). Safety and efficacy of imatinib cessation for CML patients with stable undetectable minimal residual disease: results from the TWISTER study. Blood..

[23] Modi H, McDonald T, Chu S, Yee J-K, Forman SJ, Bhatia R (2007). Role of BCR/ABL gene-expression levels in determining the phenotype and imatinib sensitivity of transformed human hematopoietic cells. Blood..

[24] Chomel J-C, Sorel N, Guilhot J, Guilhot F, Turhan AG (2012). BCR-ABL expression in leukemic progenitors and primitive stem cells of patients with chronic myeloid leukemia. Blood..

[25] Burchert A, Neubauer A, Hochhaus A (2012). Response: Too much BCR-ABL to live on, but too little BCR-ABL to die on?. Blood..

[26] Huntly BJP, Gilliland DG (2004). Blasts from the past: new lessons in stem cell biology from chronic myelogenous leukemia. Cancer Cell..

[27] Zhao C, Blum J, Chen A, Kwon HY, Jung SH, Cook JM (2007). Loss of beta-catenin impairs the renewal of normal and CML stem cells in vivo. Cancer Cell..

[28] Jamieson CHM, Ailles LE, Dylla SJ, Muijtjens M, Jones C, Zehnder JL (2004). Granulocyte-macrophage progenitors as candidate leukemic stem cells in blast-crisis CML. N. Engl. J. Med..

[29] Nieborowska-Skórska M, Ratajczak MZ, Calabretta B, Skórski T (1994). The role of c-Myc protooncogene in chronic myelogenous leukemia. Folia Histochem. Cytobiol..

[30] Williams RT, Roussel MF, Sherr CJ (2006). Arf gene loss enhances oncogenicity and limits imatinib response in mouse models of Bcr-Abl-induced acute lymphoblastic leukemia. P Natl Acad Sci Usa..

[31] Chen Y, Sullivan C, Peng C, Shan Y, Hu Y, Li D (2011). A tumor suppressor function of the Msr1 gene in leukemia stem cells of chronic myeloid leukemia. Blood..

[32] Chen Y, Hu Y, Zhang H, Peng C, Li S (2009). Loss of the Alox5 gene impairs leukemia stem cells and prevents chronic myeloid leukemia. Nat. Genet..

[33] Bose S, Deininger M, Gora-Tybor J, Goldman JM, Melo JV (1998). The presence of typical and atypical BCR-ABL fusion genes in leukocytes of normal individuals: biologic significance and implications for the assessment of minimal residual disease. Blood..

[34] Biernaux C, Loos M, Sels A, Huez G, Stryckmans P (1995). Detection of major bcr-abl gene expression at a very low level in blood cells of some healthy individuals. Blood..

[35] Gishizky ML, Johnson-White J, Witte ON (1993). Efficient transplantation of BCR-ABL-induced chronic myelogenous leukemia-like syndrome in mice. P Natl Acad Sci Usa..

[36] McLaughlin J, Chianese E, Witte ON (1987). In vitro transformation of immature hematopoietic cells by the P210 BCR/ABL oncogene product of the Philadelphia chromosome. P Natl Acad Sci Usa..

[37] Heisterkamp N, Jenster G, ten Hoeve J, Zovich D, Pattengale PK, Groffen J (1990). Acute leukaemia in bcr/abl transgenic mice. Nature..

[38] HONDA H, Oda H, Suzuki T, Takahashi T, WITTE O, Ozawa K (1998). Development of acute lymphoblastic leukemia and myeloproliferative disorder in transgenic mice expressing p210(bcr/abl): a novel transgenic model for human Ph-1-positive leukemias. Blood..

[39] Inokuchi K, Dan K, Takatori M, Takahuji H, Uchida N, Inami M (2003). Myeloproliferative disease in transgenic mice expressing P230 Bcr/Abl: longer disease latency, thrombocytosis, and mild leukocytosis. Blood..

[40] Huettner CS, Koschmieder S, Iwasaki H, Iwasaki-Arai J, Radomska HS, Akashi K (2003). Inducible expression of BCR/ABL using human CD34 regulatory elements results in a megakaryocytic myeloproliferative syndrome. Blood..

[41] Koschmieder S, Göttgens B, Zhang P, Iwasaki-Arai J, Akashi K, Kutok JL (2005). Inducible chronic phase of myeloid leukemia with expansion of hematopoietic stem cells in a transgenic model of BCR-ABL leukemogenesis. Blood..

[CR42] Foley SB, Hildenbrand ZL, Soyombo AA, Magee JA, Wu Y, Oravecz-Wilson KI (2013). Expression of BCR/ABL p210 from a Knockin Allele Enhances Bone Marrow Engraftment without Inducing Neoplasia. Cell Rep..

[43] Shachaf CM, Kopelman AM, Arvanitis C, Karlsson A, Beer S, Mandl S (2004). MYC inactivation uncovers pluripotent differentiation and tumour dormancy in hepatocellular cancer. Nature..

[44] Boxer RB, Jang JW, Sintasath L, Chodosh LA (2004). Lack of sustained regression of c-MYC-induced mammary adenocarcinomas following brief or prolonged MYC inactivation. Cancer Cell..

[45] Karlsson A, Giuriato S, Tang F, Fung-Weier J, Levan G, Felsher DW (2003). Genomically complex lymphomas undergo sustained tumor regression upon MYC inactivation unless they acquire novel chromosomal translocations. Blood..

[46] Jonkers J, Berns A (2004). Oncogene addiction: sometimes a temporary slavery. Cancer Cell..

[47] Gorgoulis VG, Vassiliou L-VF, Karakaidos P, Zacharatos P, Kotsinas A, Liloglou T (2005). Activation of the DNA damage checkpoint and genomic instability in human precancerous lesions. Nature..

[48] Braig M, Lee S, Loddenkemper C, Rudolph C, Peters AHFM, Schlegelberger B (2005). Oncogene-induced senescence as an initial barrier in lymphoma development. Nature..

[49] Bartkova J, Horejsí Z, Koed K, Krämer A, Tort F, Zieger K (2005). DNA damage response as a candidate anti-cancer barrier in early human tumorigenesis. Nature..

[50] Bartkova J, Rezaei N, Liontos M, Karakaidos P, Kletsas D, Issaeva N (2006). Oncogene-induced senescence is part of the tumorigenesis barrier imposed by DNA damage checkpoints. Nature..

[51] Halazonetis TD, Gorgoulis VG, Bartek J (2008). An oncogene-induced DNA damage model for cancer development. Science..

[52] Di Micco R, Fumagalli M, Cicalese A, Piccinin S, Gasparini P, Luise C (2006). Oncogene-induced senescence is a DNA damage response triggered by DNA hyper-replication. Nature..

[53] Serrano M, Lin AW, McCurrach ME, Beach D, Lowe SW (1997). Oncogenic ras provokes premature cell senescence associated with accumulation of p53 and p16INK4a. Cell..

[54] Lowe SW, Cepero E, Evan G (2004). Intrinsic tumour suppression. Nature..

[55] Kamijo T, Zindy F, Roussel MF, Quelle DE, Downing JR, Ashmun RA (1997). Tumor suppression at the mouse INK4a locus mediated by the alternative reading frame product p19ARF. Cell..

[56] Zindy F, Eischen CM, Randle DH, Kamijo T, Cleveland JL, Sherr CJ (1998). Myc signaling via the ARF tumor suppressor regulates p53-dependent apoptosis and immortalization. Genes Dev..

[57] Sherr CJ, Weber JD (2000). The ARF/p53 pathway. Curr. Opin. Genet. Dev..

[58] Feinstein E, Cimino G, Gale RP, Alimena G, Berthier R, Kishi K (1991). p53 in chronic myelogenous leukemia in acute phase. P Natl Acad Sci Usa..

[59] Li L, Wang L, Li L, Wang Z, Ho Y, McDonald T (2012). Activation of p53 by SIRT1 inhibition enhances elimination of CML leukemia stem cells in combination with imatinib. Cancer Cell..

[60] Kurosu T, Wu N, Oshikawa G, Kagechika H, Miura O (2010). Enhancement of imatinib-induced apoptosis of BCR/ABL-expressing cells by nutlin-3 through synergistic activation of the mitochondrial apoptotic pathway. Apoptosis..

[61] Peterson LF, Mitrikeska E, Giannola D, Lui Y, Sun H, Bixby D (2011). p53 stabilization induces apoptosis in chronic myeloid leukemia blast crisis cells. Leukemia..

[62] Lin T, Chao C, Saito S, Mazur SJ, Murphy ME, Appella E (2005). p53 induces differentiation of mouse embryonic stem cells by suppressing Nanog expression. Nat. Cell Biol..

[63] TeKippe M, Harrison DE, Chen J (2003). Expansion of hematopoietic stem cell phenotype and activity in Trp53-null mice. Exp Hematol..

[64] Akala OO, Park I-K, Qian D, Pihalja M, Becker MW, Clarke MF (2008). Long-term haematopoietic reconstitution by Trp53-/-p16Ink4a-/-p19Arf-/- multipotent progenitors. Nature..

[65] Liu Y, Elf SE, Miyata Y, Sashida G, Liu Y, Huang G (2009). p53 regulates hematopoietic stem cell quiescence. Cell Stem Cell..

[66] Krizhanovsky V, Lowe SW (2009). Stem cells: the promises and perils of p53. Nature..

[67] Jacobs JJ, Kieboom K, Marino S, DePinho RA, van Lohuizen M (1999). The oncogene and Polycomb-group gene bmi-1 regulates cell proliferation and senescence through the ink4a locus. Nature..

[68] Park I-K, Qian D, Kiel M, Becker MW, Pihalja M, Weissman IL (2003). Bmi-1 is required for maintenance of adult self-renewing haematopoietic stem cells. Nature..

[69] Mullighan CG, Williams RT, Downing JR, Sherr CJ (2008). Failure of CDKN2A/B (INK4A/B-ARF)-mediated tumor suppression and resistance to targeted therapy in acute lymphoblastic leukemia induced by BCR-ABL. Genes Dev..

[70] Komatsu N, Watanabe T, Uchida M, Mori M, Kirito K, Kikuchi S (2003). A member of Forkhead transcription factor FKHRL1 is a downstream effector of STI571-induced cell cycle arrest in BCR-ABL-expressing cells. J. Biol. Chem..

[71] Ghaffari S, Jagani Z, Kitidis C, Lodish HF, Khosravi-Far R (2003). Cytokines and BCR-ABL mediate suppression of TRAIL-induced apoptosis through inhibition of forkhead FOXO3a transcription factor. P Natl Acad Sci Usa..

[72] Naka K, Hoshii T, Muraguchi T, Tadokoro Y, Ooshio T, Kondo Y (2010). TGF-beta-FOXO signalling maintains leukaemia-initiating cells in chronic myeloid leukaemia. Nature..

[CR73] Hurtz C, Hatzi K, Cerchietti L, Braig M, Park E, Kim Y-M (2011). BCL6-mediated repression of p53 is critical for leukemia stem cell survival in chronic myeloid leukemia. J. Exp. Med..

[74] Duy C, Hurtz C, Shojaee S, Cerchietti L, Geng H, Swaminathan S (2011). BCL6 enables Ph+ acute lymphoblastic leukaemia cells to survive BCR-ABL1 kinase inhibition. Nature..

[75] Yuan H, Wang Z, Li L, Zhang H, Modi H, Horne D (2012). Activation of stress response gene SIRT1 by BCR-ABL promotes leukemogenesis. Blood..

[76] Tomasetti C, Vogelstein B, Parmigiani G (2013). Half or more of the somatic mutations in cancers of self-renewing tissues originate prior to tumor initiation. P Natl Acad Sci Usa..

[77] Melo JV, Barnes DJ (2007). Chronic myeloid leukaemia as a model of disease evolution in human cancer. Nat. Rev. Cancer..

[78] Radich JP, Dai H, Mao M, Oehler V, Schelter J, Druker B (2006). Gene expression changes associated with progression and response in chronic myeloid leukemia. P Natl Acad Sci Usa..

[79] Mullighan CG, Miller CB, Radtke I, Phillips LA, Dalton J, Ma J (2008). BCR-ABL1 lymphoblastic leukaemia is characterized by the deletion of Ikaros. Nature..

[80] Sarkisian CJ, Keister BA, Stairs DB, Boxer RB, Moody SE, Chodosh LA (2007). Dose-dependent oncogene-induced senescence in vivo and its evasion during mammary tumorigenesis. Nat. Cell Biol..

[CR81] Murphy DJ, Junttila MR, Pouyet L, Karnezis A, Shchors K, Bui DA (2008). Distinct thresholds govern Myc’s biological output in vivo. Cancer Cell..

[82] Junttila MR, Karnezis AN, Garcia D, Madriles F, Kortlever RM, Rostker F (2010). Selective activation of p53-mediated tumour suppression in high-grade tumours. Nature..

[83] Weinstein IB (2002). Cancer. Addiction to oncogenes–the Achilles heal of cancer. Science..

[84] Chomel J-C, Turhan AG (2011). Chronic myeloid leukemia stem cells in the era of targeted therapies: resistance, persistence and long-term dormancy. Oncotarget..

[85] Grant H, Jiang X, Stebbing J, Foroni L, Craddock C, Griffiths M (2010). Analysis of BCR-ABL1 tyrosine kinase domain mutational spectra in primitive chronic myeloid leukemia cells suggests a unique mutator phenotype. Leukemia..

[86] Jiang X, Saw KM, Eaves A, Eaves C (2007). Instability of BCR-ABL gene in primary and cultured chronic myeloid leukemia stem cells. J. Natl. Cancer Inst..

[87] Jiang X, Forrest D, Nicolini F, Turhan A, Guilhot J, Yip C (2010). Properties of CD34+ CML stem/progenitor cells that correlate with different clinical responses to imatinib mesylate. Blood..

[88] Graham SM, Jørgensen HG, Allan E, Pearson C, Alcorn MJ, Richmond L (2002). Primitive, quiescent, Philadelphia-positive stem cells from patients with chronic myeloid leukemia are insensitive to STI571 in vitro. Blood..

[89] Jørgensen HG, Allan EK, Jordanides NE, Mountford JC, Holyoake TL (2007). Nilotinib exerts equipotent antiproliferative effects to imatinib and does not induce apoptosis in CD34+ CML cells. Blood..

[90] Copland M, Hamilton A, Elrick LJ, Baird JW, Allan EK, Jordanides N (2006). Dasatinib (BMS-354825) targets an earlier progenitor population than imatinib in primary CML but does not eliminate the quiescent fraction. Blood..

[91] Choi YL, Soda M, Yamashita Y, Ueno T, Takashima J, Nakajima T (2010). EML4-ALK mutations in lung cancer that confer resistance to ALK inhibitors. N. Engl. J. Med..

[92] Metzelder SK, Schroeder T, Finck A, Scholl S, Fey M, Götze K, et al. High activity of sorafenib in FLT3-ITD-positive acute myeloid leukemia synergizes with allo-immune effects to induce sustained responses. Leukemia. 2012. 10.1038/leu.2012.105 22504140

